# Characteristics of Phospholipid–Immunosuppressant–Antioxidant Mixed Langmuir–Blodgett Films

**DOI:** 10.1021/acs.jpcb.2c03300

**Published:** 2022-09-06

**Authors:** Małgorzata Jurak, Klaudia Szafran, Pilar Cea, Santiago Martín

**Affiliations:** † Department of Interfacial Phenomena, Institute of Chemical Sciences, Faculty of Chemistry, Maria Curie-Skłodowska University, 20031 Lublin, Poland; ‡ Instituto de Nanociencia y Materiales de Aragón (INMA), CSIC-Universidad de Zaragoza, 50009 Zaragoza, Spain; § Departamento de Química Física, Facultad de Ciencias, Universidad de Zaragoza, 50009 Zaragoza, Spain; ∥ Laboratorio de Microscopias Avanzadas, LMA, C/Mariano Esquilor s/n, 50018 Zaragoza, Spain

## Abstract

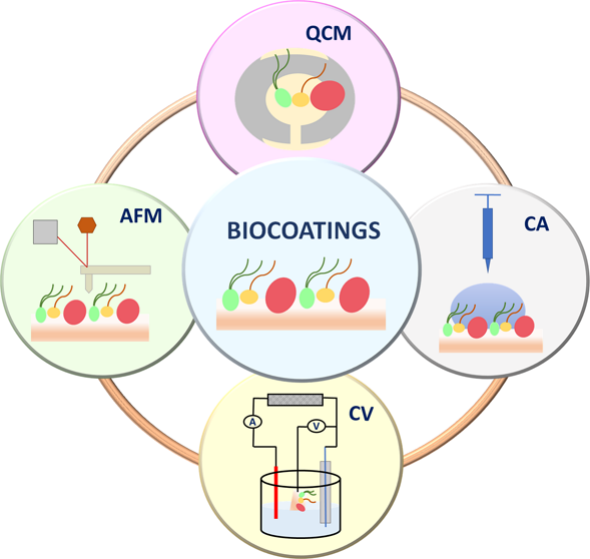

Hemocompatibility is one of the major criteria for the successful cardiovascular applicability of novel biomaterials. In this context, monolayers of certain biomolecules can be used to improve surface biocompatibility. To this end, biocoatings incorporating a phospholipid (1,2-dioleoyl-*sn*-glycero-3-phosphocholine, DOPC), an immunosuppressant (cyclosporine A, CsA), and an antioxidant material (lauryl gallate, LG) were fabricated by depositing Langmuir films onto gold or mica substrates using the Langmuir–Blodgett (LB) technique. These LB monolayers were thoroughly characterized by means of quartz crystal microbalance (QCM), atomic force microscopy (AFM), cyclic voltammetry (CV), and contact angle (CA) measurements. The obtained results indicate that the properties of these LB films are modulated by the monolayer composition. The presence of LG in the three-component systems (DOPC–CsA–LG) increases the molecular packing and the surface coverage of the substrate, which affects the wettability of the biocoating. From the different compositions studied here, we conclude that DOPC–CsA–LG monolayers with a DOPC/CsA ratio of 1:1 and LG molar fractions of 0.50 and 0.75 exhibit improved surface biocompatible characteristics. These results open up new perspectives on our knowledge and better understanding of phenomena at the biomaterial/host interface.

## 1 Introduction

Cardiovascular diseases are the most common cause of death worldwide. Arterial stenting has emerged as a promising minimally invasive stent-based therapy. Since most cardiovascular stents are built of stainless steel and metal alloys, corrosion may play a key role in the medium- and long-term results of implantation.^1,2^ The corroding metals release toxic metal ions into the adjacent tissues and blood, which alter the chemistry near the implant and affect its integrity and mechanical properties. As a response to these extraneous toxic materials in the body, local immune response leading to intimal hyperplasia and in-stent restenosis occurs.^2−4^ Therefore, the reduction of the restenosis and thrombosis effects is the driving force for numerous innovative strategies for stent surface conditioning.

A diversity of materials has been employed as a protective coating for cardiovascular metallic stents.^2^ The principal function of such coatings is to improve the biocompatibility by preventing ion release from the stent surface. It is well known that gold is corrosion-resistant; additionally, gold exhibits fluoroscopic visibility^5,6^ and adapts well in the human body.^7,8^ Gold-coated stainless steel stents were extensively explored in the coronary stenting methodology owing to gold inertness, small platelet adhesion, and low thrombus formation.^6,9^ Despite these promising properties, the clinical trials did not prove to be fully satisfactory.^8,10^ The gold-coated stents led to great rates of restenosis^6,11^ and allergy reactions due to dissolution of the Au coating, ion diffusion, and gold concentration in the blood.^12^ An additional protective layer on top of the gold coating can be used with the purpose of diminishing thrombogenicity and enhancing stent biocompatibility. Such an additional layer will screen the stent surface from the attack of environmental factors capable of disturbing the stent integrity and its function.^8,10^ In this context, the conception of employing biocompatible coatings is very appealing and has emerged as a hot research area in recent years.^8,13−16^ Therefore, the fabrication of a coating for modifying surface implants and, more specifically, to design a biocompatible system that can be used in tissue engineering, mainly in the cardiovascular system, is a current topic of interest. Biocoatings should accomplish the two following characteristics: (i) integrity of the coating should be ensured^8,17^ for reliability and safety of the stent device and (ii) uniformity and stability of the coating with well-defined physicochemical and adhesive properties are required to avoid localized corrosion due to local defects.

Adsorption of biological molecules onto the gold-coated metal stent surface can ensure the chemical stability by controlling the rate of corrosion, provide nontoxicity and biodegradability, and also serve as platforms for drug delivery.^8,18^ Additionally, administration of certain drugs with anti-inflammatory, immunosuppressive, antithrombogenic, and antiproliferative effects^8,19^ may be of interest to reduce the immunoresponse. However, ineffective systemic use of such drugs can result from too low concentrations in the site of stent implantation.^20,21^ For this reason, the application of those drugs in the implant proximity may result in a more effective approach.

**Figure 1:**
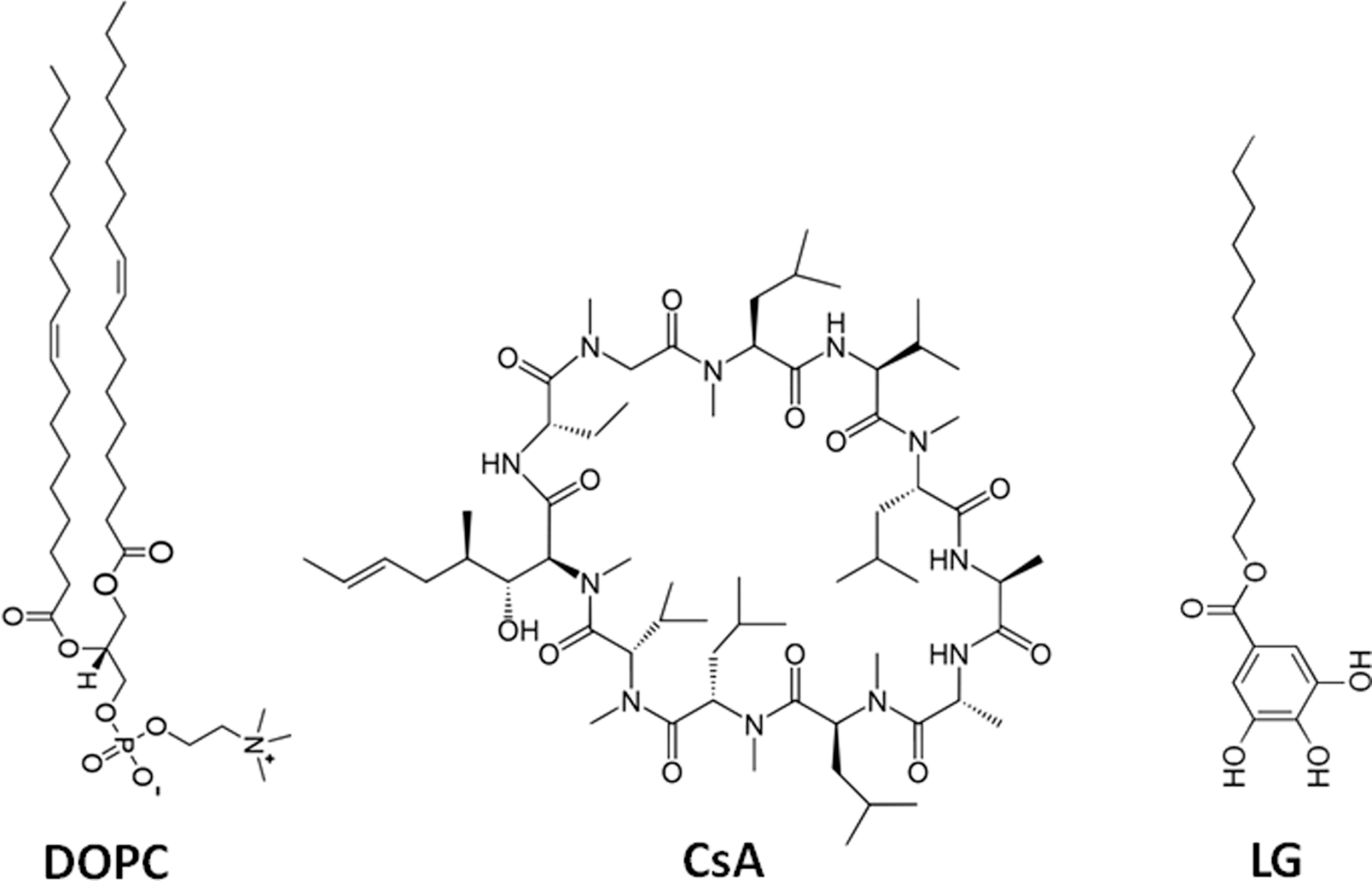
Chemical structures of 1,2-dioleoyl-*sn*-glycero-3-phosphocholine (DOPC), cyclosporine A (CsA), and lauryl gallate (LG) used in this study.

Phosphatidylcholine, PC (Figure 1), is the most abundant lipid in eukaryotic cells and is a major neutral phospholipid in membrane blood cells. Importantly, PC can act as a link between the artificial material and living systems, thus contributing to an increase in implant surface biocompatibility. A representative compound in the PC family is dioleoylphosphatidylcholine (DOPC), which possesses two C18 hydrocarbon chains with one double bond in the middle of each of them. The chain unsaturation yields a low main phase transition temperature. This ensures the liquid crystalline state at room and physiological temperature, reflecting the fluid state of natural membranes. For these reasons, DOPC is one of the most commonly used unsaturated phospholipids in model membrane studies.^22−24^

Furthermore, PC has been probed as an effective drug-eluting platform,^25^ particularly for the above-mentioned active drugs;^8,19^ PC also facilitates the drug loading and the controlled release in the target sites, providing an adequate concentration of a drug locally without adverse systemic effects.^10,21^ Importantly, PC forms a passive barrier against exposure of the stent material to the bloodstream and strengthens its hemocompatibility.^2,26^ For these reasons, PC may be a relevant material in implant coatings.

In addition to the incorporation of PC on implant coatings, we also focus our attention here on cyclosporine A (CsA, Figure 1). CsA is an immunosuppressive drug, whose poor water solubility makes it ideal for surface coatings. Since CsA inhibits the signal transduction pathways of T-cell receptors, it is the most frequent drug used in organ transplants and implant surgery to prevent rejection as well as in the treatment of autoimmune diseases.^27^ However, a side effect of CsA is the generation of reactive oxygen species, which, in the absence or reduced repair mechanisms of the cell, leads to biomolecule damage processes, including lipid oxidation, that disable structures and functions of some organs, such as the kidney, liver, and heart.^28^ These processes are directly responsible for the nephro-, hepato-, and cardiotoxicity of the drug. Additionally, excessive production and accumulation of oxygen free radicals is a major source of damage to the inflamed tissues and implanted materials resulting in their rejection.

To minimize the undesirable/adverse effects of CsA, the application of antioxidative effects of polyphenols capable of quenching toxic free radicals is one of the possible solutions. The protective role of various antioxidants was revealed as regard to the CsA-induced toxicity.^27,28^ Polyphenols possess the ability to bind directly to target proteins and peptides.^29,30^ Their antioxidant action toward free radicals generated by cellular metabolism or in response to exterior factors is due to the redox properties, wherein the hydrogen-atom or single-electron transfer and metal chelation are the main mechanisms of the antioxidant capability.^29,30^ A representative polyphenol is lauryl gallate (LG, Figure 1). LG with multiple −OH and >C=O groups, besides scavenging the free radicals and reacting oxygen species (ROS), can offer several available sites for stable metal complexation. When entrapped in complexes, metals cannot participate in the reactions producing free radicals becoming less reactive or even inactive,^29,30^ preventing the side effects of corrosion. Thus, the use of such an antioxidant has numerous advantages.

We have previously demonstrated the existence of repulsive interactions between DOPC and CsA with partial miscibility of the two components that result in the disruption of the order of the acyl chains.^31^ Importantly, the addition of the antioxidant LG, which interacts competitively with both DOPC and CsA, increases the miscibility of components and improves the film stability.^31^ The objective of this work is to extend our previous investigation on the miscibility of DOPC, CsA, and LG in two ways by demonstrating that (1) mixed Langmuir films of DOPC–CsA–LG (Figure 1) can be transferred by the Langmuir–Blodgett (LB) technique to modify mica and/or gold substrates, resulting in high-quality films with enhanced physicochemical properties and (2) the composition of these mixed DOPC–CsA–LG LB films can be optimized for further control of the final physicochemical properties of these selected biocoatings. This fundamental research is essential for the design and development of more efficient medicinal strategies against CsA adverse effects and for stent biocompatibility improvement.

## 2 Experimental Section

### 2.1 Materials

1,2-Dioleoyl-*sn*-glycero-3-phosphocholine (DOPC, ≥99%, Sigma), cyclosporine A (CsA, ≥99%, Alfa Aesar), and lauryl gallate (LG, ≥99%, Aldrich) were used as received. Stock solutions (1 mg mL^–1^) of these compounds were prepared in chloroform/methanol (4:1, v/v) solutions (chloroform, Macron Fine Chemicals, 99.8%; methanol, Fluka, ≥99.9%). Then, by mixing appropriate volumes of the stock solutions, the binary (DOPC–CsA 0.50) and ternary (DOPC–CsA–LG 0.25, 0.50, 0.75) mixtures of different molar ratios of components were prepared (keeping a DOPC/CsA ratio of 1:1, where the numbers denote the molar fraction of the LG component). All solutions were stored in dark glass bottles to protect them from light-induced damage.

### 2.2 Methods

Langmuir and Langmuir–Blodgett (LB) monolayers were fabricated in a KSV NIMA LB trough with dimensions 580 × 145 mm^2^, located in a semiclean room at constant temperature (20 ± 1 °C). Additionally, the trough was placed in a light-tight chamber. Millipore Milli-Q water (18.2 MΩ cm) was used as a subphase. The surface pressure (π) was determined using a Wilhelmy paper-plate pressure sensor. After spreading the solution using a Hamilton syringe, the system was left for 10 min for solvent evaporation before starting the compression of the film with the trough barriers moving at 29 cm^2^ min^–1^. Langmuir monolayers were deposited onto mica, gold-on-quartz, gold-on-glass, or gold-on-mica substrates at 10 mN m^–1^ by the vertical dipping method (emersion) at a rate of 5 mm min^–1^. Three independent series of LB deposition were carried out. The experimental error is represented by the standard deviation of transfer ratio (TR) values.

To avoid the loss of molecules, the transference of the monolayer onto the solid supports was performed immediately after reaching the target pressure. To prevent multilayer formation and therefore to ensure the presence of all of the components in the mixed monolayers studied here, a transference surface pressure significantly lower than that of the component with the lower collapse surface pressure must be chosen. In our case, such surface pressure of transference was 10 mN m^–1^. This value was selected taking into account the collapse surface pressure of the CsA monolayer (23.1 mN m^–1^) that is considerably lower than that of DOPC (43.3 mN m^–1^) and LG (45.9 mN m^–1^).^31^

Mica sheets were provided by Continental Trade (Poland) and were cleaved with adhesive tape prior to be used. Gold-on-glass substrates (purchased from Arrandee, Germany) were flame-annealed using literature procedures at approximately 800–1000 °C with a Bunsen burner flame to obtain atomically flat Au(111) terraces^32^ immediately prior to be used. Gold-on-mica was received from Georg Albert PVD Beschichtunben (Germany) and, just before use, it was rinsed gently with ultrapure water.

Quartz crystal microbalance (QCM) measurements were carried out using a Stanford Research Systems instrument with AT-cut quartz crystals (resonant frequency of 5 MHz) patterned with circular gold electrodes on both sides. The gold surface of the QCM resonator was immersed completely in the subphase prior to LB deposition. The obtained surface coverage (μg cm^–2^) shows the averaged data determined from at least three independent measurements.

Atomic force microscopy (AFM) images were obtained with a Bruker Multimode 8 microscope with a Nanoscope V control unit under ambient air conditions at a scan rate of 1 Hz using the tapping mode. RTESPA-150 AFM tips were purchased from Bruker (90–210 kHz resonant frequency, 5 N m^–1^ spring constant, and a nominal tip radius of 8 nm). The average height deviations of the surface are given by the root mean square (RMS) roughness parameter.

An Autolab potentiostat (Eco Chemie) with a standard three-electrode cell (reference electrode: Ag|AgCl|satd KCl, counter electrode: Pt sheet, and working electrode: an Au(111) substrate covered with or without an LB film) was used to perform cyclic voltammetry (CV) experiments. A 0.1 M KCl solution was used as an electrolyte. Taking into account the active substrate surface area (0.05 cm^2^), the current density (in μA cm^–2^) was determined for both bare and coated gold samples. For each experiment, two voltammetric cycles were recorded with no significant differences between them.

Contact angle (CA) measurements were acquired by means of the optical tensiometer (Attension Theta Lite) using the sessile drop method. The contact angle was determined by placing a 5 μL drop of liquid dispensed from a syringe onto the material surface. Then, a camera with a goniometer captured an image of the drop on the surface and, based on the data analysis, the advancing contact angle was determined. The obtained contact angle images are shown in the Supporting Information (Figures S5 and S6). The measurements were repeated on different regions of the given surface in three separate experiments, taking a reading on the left and right side of the droplets. Thus, the contact angles were calculated as the arithmetic mean of the measured values (for up to 15 drops), and the standard deviation was evaluated to represent the experimental error.

## 3 Results and Discussion

Since the surface of the biomaterial first comes into contact with the biological cells or fluids, its characteristics should be carefully examined. Regarding the biocompatibility improvement, the implant surface can be modified by covering it with a molecular layer, which will also serve as a platform for drug loading.^8^ Additionally, coatings for stents should provide nontoxicity, proper surface topography, and roughness as well as wettability to regulate adsorption of biological molecules and cells in the human body.

A detailed characterization of Langmuir monolayers at the air–water interface of DOPC, CsA, and LG in one-, two-, and three-component systems was carried out in our previous work.^31^ The study was based on analyzing π–*A* and Δ*V*–*A* isotherms as well as the compressibility modulus, excess surface area, and Gibbs energy of mixing and dipole moment values at the air–water interface. The data consistently showed that the addition of LG molecules to the DOPC–CsA monolayer induced more attractive interactions between components in the ternary systems, becoming thermodynamically more stable Langmuir films. Three different multicomponents DOPC–CsA–LG 0.25, 0.50, 0.75 films (keeping a DOPC/CsA ratio of 1:1, where the numbers denote the molar fraction of the LG component) would be suitable for the preparation of a stable cover for the forthcoming deposition on an implant material surface.

Therefore, here, we carry out the study and characterization of LB films formed by transferring the corresponding Langmuir film (DOPC–CsA 0.50 or DOPC–CsA–LG 0.25, 0.50, 0.75) onto mica or gold substrates by the vertical method at the transference surface pressure of 10 mN m^–1^. Since the transfer pressure should be below the collapse surface pressure, a transfer pressure of 10 mN m^–1^ has been chosen to avoid the collapse of some of the components and to ensure the presence of all components in the monolayer. For these reasons, the transfer surface pressure is below the collapse pressure value of the CsA monolayer, which is the lowest one of all films studied.^31^ Additionally, at this surface pressure, we assure that the monolayers are in a liquid-expanded phase, reflecting the fluid state of the natural membranes, as it was demonstrated previously,^31^ through compression modulus (*C*_S_^–1^) values. The *C*_S_^–1^ values of 48, 66, and 30 mN m^–1^ at the surface pressure of 10 mN m^–1^ for the DOPC, CsA, and LG films, respectively, were obtained, revealing that CsA forms the less flexible films, in agreement with the lowest collapse surface pressure obtained for this monolayer, while the *C*_S_^–1^ values of 41, 45, and 41 mN m^–1^ were obtained for DOPC–CsA–LG 0.25, 0.50, and 0.75, respectively, revealing the DOPC–CsA–LG 0.50 system to be less fluid (Table S1).

Stability of the monolayers and the transfer process on mica and on gold substrates at the surface pressure of 10 mN m^–1^ are presented in Table 1 and in the Supporting Information.

**Table 1:** Experimental Molecular Area (*A*_e_) Determined from the QCM Data, Theoretical Molecular Area (*A*_t_), and Molecular Area Determined from the π*–A* Isotherms at 10 mN m^–1^ (*A*_i_) for the Indicated Single, Binary, and Ternary Monolayers^a^^,^^b^

monolayer	*A*_e_ (Å^2^)	*A*_t_ (Å^2^)	*A*_*i*_ (Å^2^)	gold	mica
DOPC	90.9	67.0	78.4	0.8 ± 0.1	0.8 ± 0.3
CsA	172.4	182.0 v	210.1	1.1 ± 0.2	1.0 ± 0.1
		374.0 h			
DOPC–CsA 0.50	125.0		156.2	1.3 ± 0.1	1.0 ± 0.2
DOPC–CsA–LG 0.25	104.2		116.9	1.2 ± 0.2	0.9 ± 0.3
DOPC–CsA–LG 0.50	47.6		88.2	1.8 ± 0.2	1.1 ± 0.3
DOPC–CsA–LG 0.75	52.6		63.3	1.1 ± 0.2	0.9 ± 0.3
LG	71.4	24.0	37.5	0.9 ± 0.1	0.9 ± 0.2

a Transfer ratio (TR) values for the deposition process on gold and mica are also included, where Δ*A*_m_ is the experimental monolayer surface area decrease and *A*_s_ is the substrate coated area.

b Note: v: vertical orientation; h: horizontal orientation of a molecule.

### 3.1 Quartz Crystal Microbalance (QCM) Measurements

The surface coverage at the surface pressure of 10 mN m^–1^ for the single (DOPC, CsA, LG), binary (DOPC–CsA 0.50), and ternary (DOPC–CsA–LG 0.25, 0.50, 0.75) monolayers was quantitatively determined through the difference in the QCM resonator frequency (Δ*f*) before and after the LB film formation process. The Sauerbrey equation was then applied to determine the corresponding surface coverage^33^
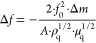
1where *f*_0_ is the fundamental resonant frequency of ca. 5 MHz, Δ*m* is the change in mass, *A* is the electrode area, μ_q_ is the shear modulus (2.95 × 10^11^ dyn cm^–2^), and ρ_q_ is the density of quartz (2.65 g cm^–3^).

**Figure 2:**
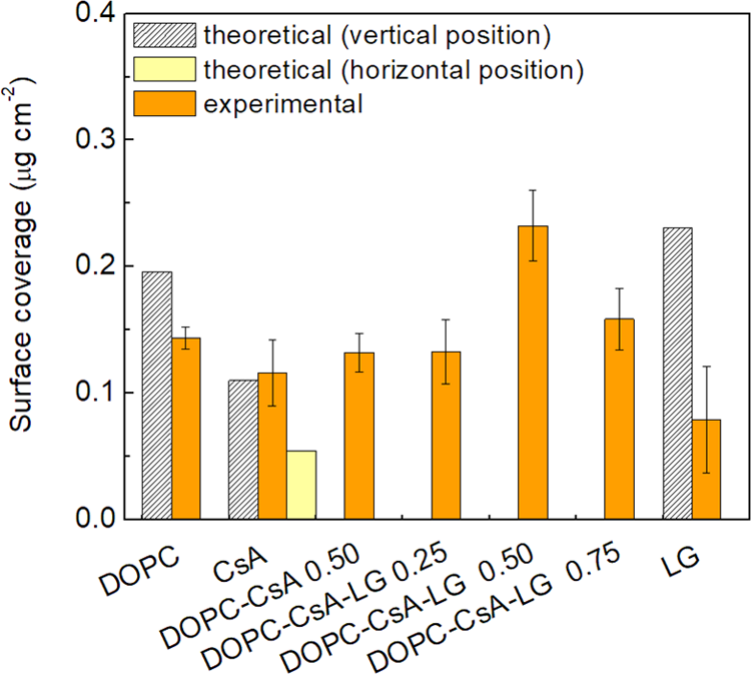
Surface coverage for the indicated single, binary, and ternary monolayers as well as the theoretical surface coverage determined taking into account a vertical or horizontal (CsA) arrangement of the molecules.

Figure 2 shows the determined surface coverage expressed in μg cm^–2^ for the different monolayers considered here. In addition, the surface coverage in molecules cm^–2^, which was determined by considering the averaged molecular weight of the components, attending their molar fraction in the mixture is presented in the Supporting Information (Figure S4). For one-component monolayers, the experimental surface coverage was compared with the theoretical one calculated on the basis of a limiting area of DOPC (67 Å^2^ molecule^–1^),^34,35^ a molecular area in both vertical (182 Å^2^ molecule^–1^) and horizontal (374 Å^2^ molecule^–1^) position for CsA,^36^ and a molecular model for the vertically arranged molecule for LG (24 Å^2^ molecule^–1^; Spartan 08 V 1.2.0). The theoretical molecular area (*A*_t_) is shown in Table 1. The experimental surface coverage values for the pure films of DOPC and LG are smaller than the theoretical ones calculated for the molecules arranged in the most vertical orientation they can reach with respect to the substrate surface (Figure 2). This observation may be attributed to a number of reasons, including deficient deposition of the films and uncovered areas (which seems not to be the case if one considers the transfer ratios and the AFM images shown below), loosy films in which the molecules are not tightly packed, or the presence of molecules arranged in a tilt orientation toward the substrate surface. Accordingly, the obtained results are reasonable taking into account that all monolayers are transferred in a fluid phase (at 10 mN m^–1^).^31^ It should be emphasized here that the presence of double bonds in the *cis*-conformation of the oleoyl DOPC chains as well as the relatively large (bulky) polar pyrogallol groups of LG prevent fully vertical orientation for these molecules on compression. Surprisingly, the experimental surface coverage is even slightly higher than the theoretical one for CsA in a vertical position, which indicates that some excess CsA molecules can be deposited on the substrate surface. This hypothesis is further confirmed by the transfer (coverage) ratio values shown below.

The experimental surface coverage for the binary DOPC–CsA 0.50 monolayer is intermediate between that of DOPC and CsA (Figure 2), as expected, while the insertion of LG to the binary DOPC–CsA monolayer increases the surface coverage. Such an increased surface coverage of the DOPC–CsA–LG monolayers with respect to the DOPC–CsA ones is tentatively attributed here to the contribution of specific attractive interactions between components, resulting in more tightly packed monolayers. The largest surface coverage is obtained for the DOPC–CsA–LG 0.50 monolayer, which is consistent with the strongest attraction between molecules and their tightest packing at the air–water interface.^31^

Moreover, from the experimental surface coverage, the area per molecule (*A*_e_) determined from the QCM results, and the area per molecule at the transfer surface pressure (*A*_*i*_), the transfer (coverage) ratio () was estimated. TR provides quantitative information about the transfer process. Table 1 summarizes the indicated parameters.

**Figure 3:**
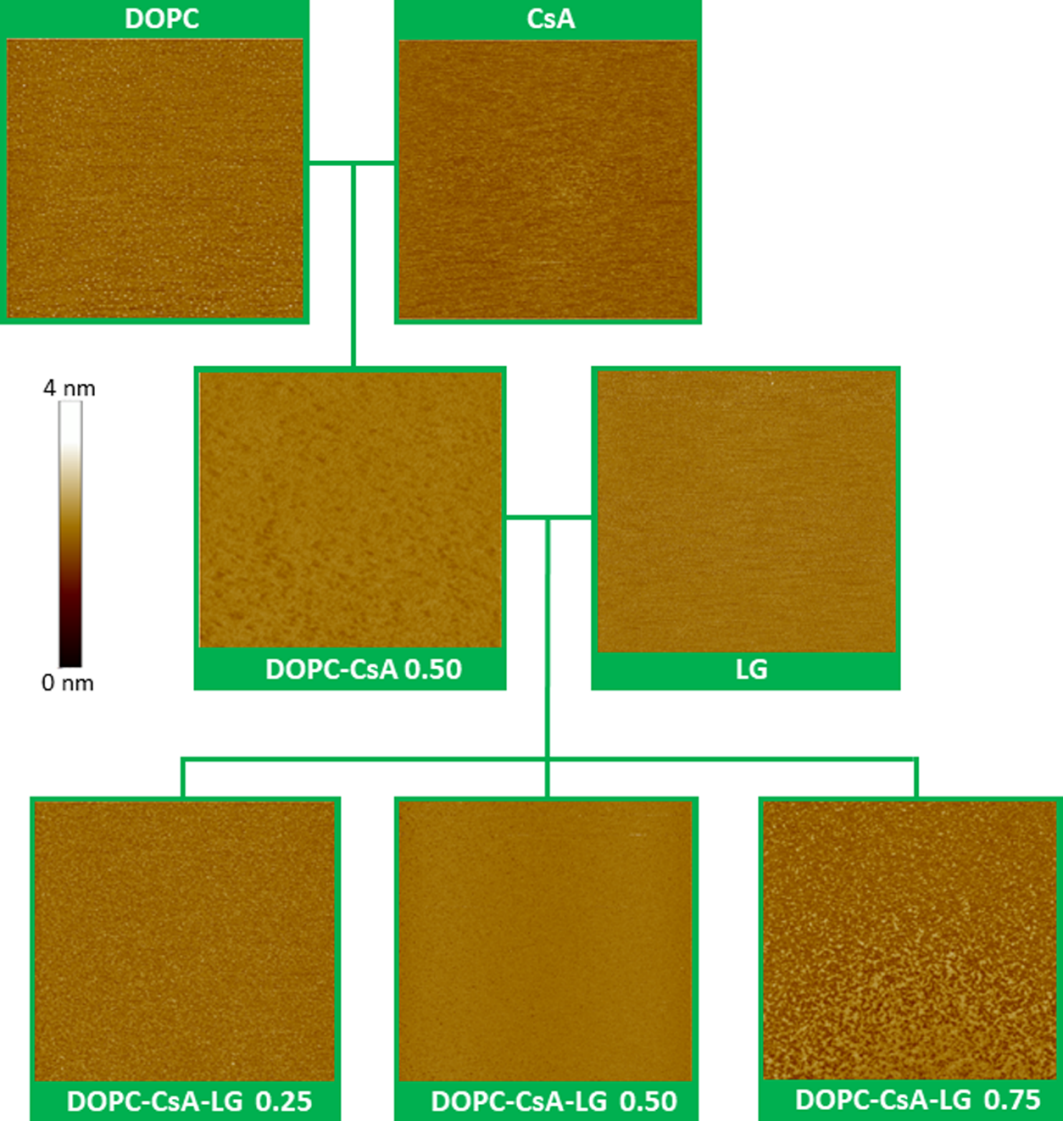
AFM images (3 × 3 μm^2^) for the indicated one-layer LB films transferred at 10 mN m^–1^ onto mica.

**Figure 4:**
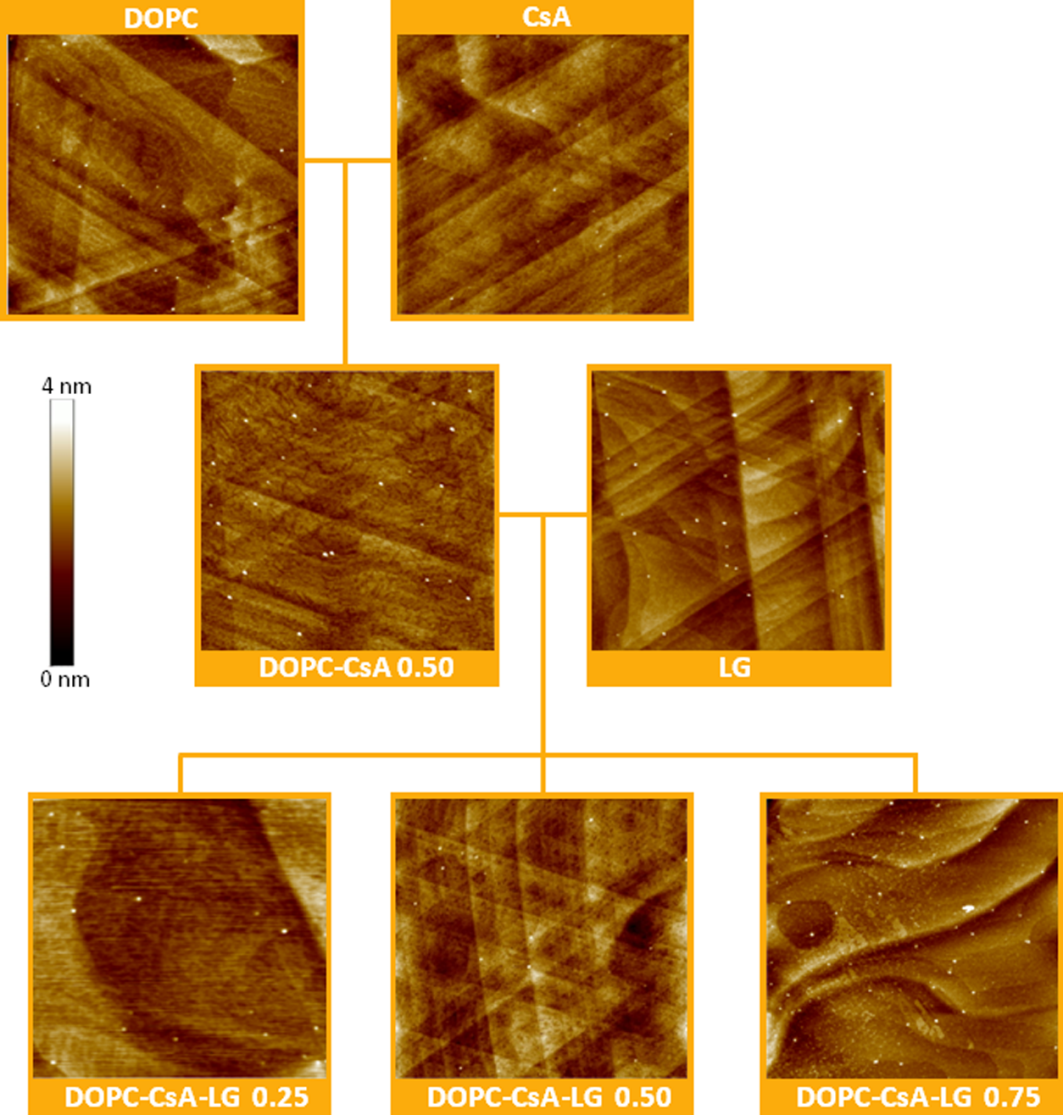
AFM images (2 × 2 μm^2^) for the indicated one-layer LB films transferred at 10 mN m^–1^ onto gold-on-mica substrates.

The molecular areas estimated from the QCM data (*A*_e_) differ from those determined from the π–*A* isotherms at the transfer pressure (*A*_*i*_). These differences significantly depend on the film composition. For DOPC and LG films, the *A*_e_ value (after deposition) is larger, while for the other monolayers, it is smaller than *A*_*i*_ (before deposition). For the DOPC film, this result is attributed here to the desorption of molecules from the air–water interface as revealed by the instability of the film (Figure S1); meanwhile, for a LG film, this observation is probably a consequence of a reorganization of the molecules upon the transference process toward a more tilted orientation of the molecules due to a weaker interaction of these with the gold substrate. Importantly, despite the loose packing of the monolayers (liquid-expanded phase) on water, the chosen surface pressure of transference is high enough to ensure sufficient cohesion in the monolayers during transfer to the solid substrate as will be demonstrated below by AFM, CV, and contact angle measurements. On the contrary, TR values greater than unity point out deposition enhancement since more molecules per unit area are being transferred onto the substrate compared to the water interface. This is particularly noticeable for DOPC–CsA–LG 0.50 (TR = 1.8 and 1.1 for a gold and mica substrate, respectively). As the presence of a great number of aggregates or even the formation of multilayers, which could justify TR values higher than 1, is discarded as revealed by the AFM images shown in Figures 3 and 4, it can be attributed to a condensing effect of the monolayer upon deposition due to a different balance of forces between the molecules when they are disposed onto different surfaces (gold or mica vs water).

Therefore, TR values higher than 1 can be attributed to a reorganization of the molecules during the transference process toward more compact films, which could be due to a stronger interaction of the molecules with the substrate respect to water as well as to an increase of the strong attractive interactions between the molecules forming the mixed monolayers through H-bonds and Lifshitz–van der Waals forces. Hence, due to the substrate nature (specific surface interactions) and the subsequent changes in the intermolecular interactions induced by those interfacial interactions, the molecules could adopt a more vertical orientation.

When a mica substrate was used, the transfer ratio (TR) values are determined as a decrease in the monolayer surface area on the subphase (Δ*A*_m_) divided by the substrate coated area (*A*_s_) as shown in Table 1 and in the Supporting Information (Figure S3 and Table S1). It should be emphasized that due to the instability of the monolayers (Figure S1), the Δ*A*_m_ values employed here to calculate the transfer ratio are corrected for the decrease of the monolayer surface area related to the loss of molecules in the time needed to transfer the film. Specifically, to compensate for the desorption of the molecules and keep the surface pressure constant when transferring, the barriers generate further compression. Consequently, a higher decrease in the monolayer surface area on the subphase than it actually accompanies the deposition process onto the solid support is observed resulting in an overestimation of TR values determined from Langmuir trough software (Figure S3). However, taking into account the loss of area per molecule with time at 10 mN m^–1^ during the entire deposition process onto mica, it allows correcting the monolayer surface area decrease (Δ*A*_m_). As a result, the obtained TR values get closer mostly to those for LB films on gold (Table 1). In contrast, the QCM coverage data do not include the area loss due to the film instability, but only the area per molecule at the target surface pressure in the π–*A* isotherm just before the transfer process starts and the area per molecule gained from the surface coverage in the gold-on-quartz substrate. This explains the differences in TR values determined by both methods.

It is worth emphasizing that atomic smoothness of mica along with high surface charge density^37^ allow for strong adhesion of transferred molecules, leading to compact film formation. Since the mica surface is known to be negatively charged in contact with water, molecule adsorption is driven by electrostatic forces and/or hydrogen bonding between the polar head groups in the monolayer and the solid surface. These processes can be enhanced by positive values of the surface potential changes (Δ*V*) of the monolayers^31^ listed in Table S1. In line with the above expectation, one can notice values of TR on mica close to unity (Table 1). In contrast, it should be highlighted that even the negative potential of the LG monolayer does not hinder its deposition on mica. This suggests that adhesion to the substrate surface is mainly due to the contribution of the terminal groups immersed in water, not necessarily potential-generating ones. It is likely that hydroxyl groups immersed in the subphase do not contribute to the negative surface potential of LG, which is caused principally by the polarization of carbonyl groups (carbonyl dipoles) near the hydrophobic region of the monolayer.^31^ Hence, participation of the polar −OH groups of the pyrogallol moiety, with partial positive charge accumulated on the H atoms, in interactions with the negatively charged mica determines the transference efficiency, which can be also favored by the LG ability to intermolecular hydrogen bond network formation^38^ (Table 1).

The above findings indicate that the surface potential values of the floating monolayers do not significantly affect the transfer process under the experimental conditions used. Nevertheless, the highest TR values on both substrates (gold and mica) were obtained for the DOPC–CsA–LG 0.50 monolayer with the lowest positive potential value (224 mV, Table S1).

### 3.2 Atomic Force Microscopy (AFM)

As is well known, the surface topography of the implant significantly affects the cell growth and multiplication.^2,39,40^ Therefore, by modifying the topography of the implant surface, its physical and chemical properties can be changed, improving the response of endothelial tissues to the stent implantation.^41^ In addition, the surface roughness defines the stent area contacting with the endothelium and it also determines the amount of adsorbed proteins.^2,42^ For these reasons, the topography of the single (DOPC, CsA, LG), binary (DOPC–CsA 0.50), and ternary (DOPC–CsA–LG 0.25, 0.50, 0.75) monolayers was studied using an atomic force microscope (AFM). Two substrates were used as support of the LB films: gold-coated mica sheets (gold as an outermost layer of stent material) and freshly cleaved mica sheets. Besides being the preferred substrate for AFM imaging, mica is a perfect support for LB films due to its atomically smooth surface and hydrophilic character, which ensure that the fragile structure of the interfacial film practically remains intact despite the transfer procedure. Therefore, this allows evaluating the influence of the used substrate in the topography of the LB films.

Figure 3 shows the representative AFM images for one-layer LB films of DOPC, CsA, LG, DOPC–CsA 0.50, and DOPC–CsA–LG 0.25, 0.50, 0.75 (numerals denote the molar fraction of the last component) transferred at 10 mN m^–1^ onto mica. DOPC, CsA, and LG form homogeneous films onto the mica substrate, which is confirmed by the low values obtained for the root mean square (RMS) surface roughness, 0.29, 0.34, and 0.19 nm for DOPC, CsA, and LG, respectively. The mixed DOPC–CsA 0.50 LB film shows the presence of certain areas with different heights, which can be attributed to a different tilt of the molecules with respect to the substrate giving an RMS value of 0.44 nm. This result is consistent with the existence of repulsive interactions (partial miscibility) between DOPC and CsA molecules, as it was previously observed in Langmuir films.^31^ For the DOPC–CsA–LG 0.25 ternary system, a homogeneous film is obtained (RMS 0.04 nm). This observation can be interpreted in terms of the LG affinity for both DOPC and CsA, which is revealed in attractive DOPC–LG and CsA–LG interactions in the binary systems. Therefore, when added to the DOPC–CsA mixture with the repulsive nature of interactions, LG is expected to act as a linker between both compounds that favor miscibility through hydrogen bonding and Lifshitz–van der Waals forces.^31^ As the amount of LG increases (DOPC–CsA–LG 0.50), a more homogeneous film is obtained (RMS 0.03 nm), indicating a better miscibility between the components in this mixing ratio, which is in good agreement with the thermodynamic parameters obtained at the air–water interface.^31^ Nevertheless, the DOPC–CsA–LG 0.75 system exhibits a less homogeneous topography with clearly visible domains, RMS 0.20 nm (Figure 3). At this LG proportion, a suitable structural arrangement is not ensured and, thus, the lack of spatial matching and the different strength of the DOPC–LG and CsA–LG interactions can lead to the formation of domains.^31^

Figure 4 shows AFM images of one-layer LB films transferred at 10 mN m^–1^ onto gold-on-mica substrates (gold as a stent material) for all systems studied here. The topography of these LB films transferred on gold-on-mica even when closely following the topography of the entirely covered underlying substrate and characteristic features of gold-on-mica, such as steps and terraces, remains visible through the LB film is very similar to the one observed for LB films deposited onto mica for each one of the systems (Figure 3), although with the presence of a few local collapses (brightest spots) when the gold-on-mica substrate is used. In addition, the RMS roughness of the gold-on-mica-supported LB films is slightly higher than that of the monolayers on mica, as the steps and terraces of the gold-on-mica substrates visible through the monolayer influence on this. RMS values of 0.35, 0.45, 0.33, 0.56, 0.32, 0.29, and 0.40 nm for DOPC, CsA, LG, DOPC–CsA 0.50, and DOPC–CsA–LG 0.25, 0.50, and 0.75, respectively, are obtained. These observations reveal that the physicochemical features of the substrate surface can play an important role in the process of monomolecular film formation and its properties. A different substrate can provoke a different packing of the deposited molecules. Remarkably, in both cases, the DOPC–CsA–LG 0.50 system results in the most homogeneous film.

The materials employed in cardiovascular applications should meet the requirements for hemocompatibility. The surface of a natural vessel in contact with blood is not smooth as it contains micrometer-sized corrugated grooves with nanoprotrusions at the top of the projections.^43^ Additionally, the wall surface is a binding site for plasma proteins, which affects the platelet binding and can induce thrombosis.^44^ When the stent surface roughness increases, the activation of platelets and their further aggregation may lead to the thrombus formation.^2^ Meanwhile, very smooth surfaces largely prevent stent in contact with human blood^45^ from corrosion and also avoid platelet adhesion and further aggregation.^46^ Thus, for the stent applications, surface modification should be aimed at obtaining a smooth surface, free or practically free of defects and contaminations.^21^ The pure as well as the binary and ternary LB monolayers studied here have been shown to result in very flat surfaces, which opens the path for their use in surface coatings of implant materials.

### 3.3 Cyclic Voltammetry (CV)

**Figure 5:**
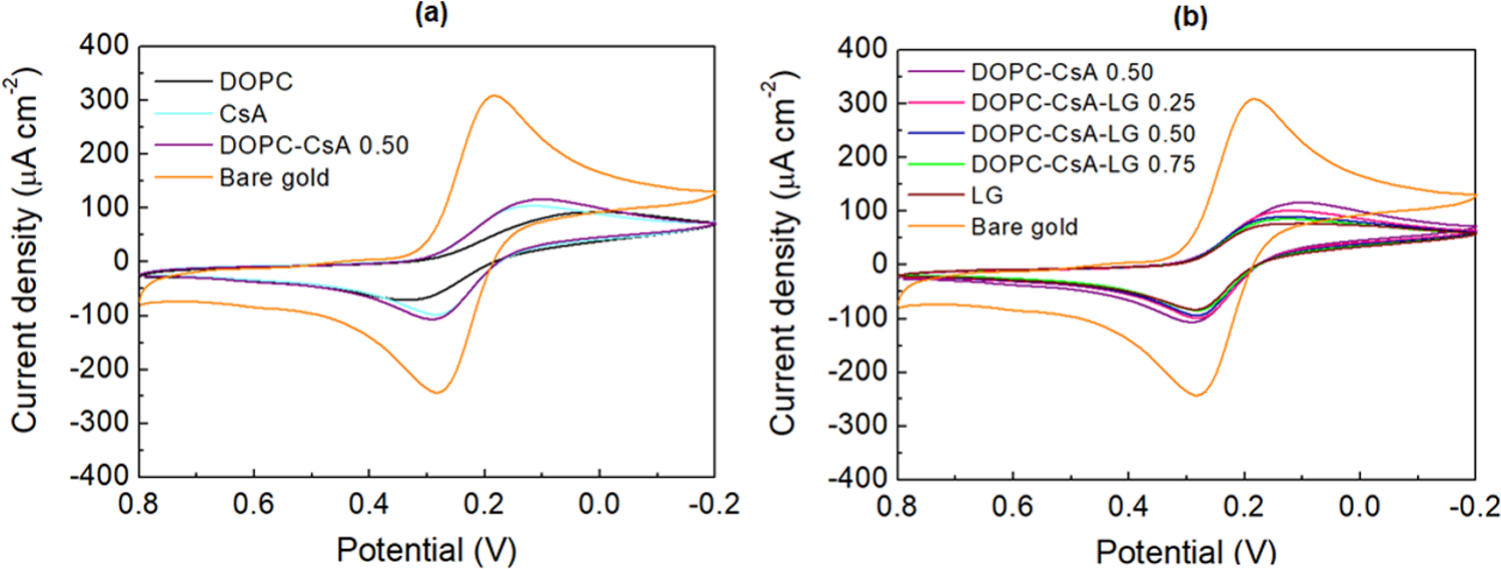
Cyclic voltammograms obtained for a bare gold electrode and modified gold electrodes with DOPC, CsA, LG, and the indicated binary and ternary systems. Scan rate was 0.1 V s^–1^, and the initial scan direction was negative. The reference electrode was Ag|AgCl|satd KCl, and the counter electrode was a Pt sheet.

In addition to the AFM images, an indirect evaluation of defect densities in the LB films may be conveniently obtained by cyclic voltammetry (CV).^47^ Here, we have studied the electron transfer reaction between a redox couple in an electrolyte solution (0.1 M KCl aqueous solutions containing 1 mM K_3_[Fe(CN)_6_] as a redox probe) and the underlying gold electrode^48^ modified with the different monolayers studied here. Figure 5 shows the cyclic voltammograms for the indicated LB film-modified gold electrodes in the 0.8 to −0.2 V range. In addition, Table 2 gathers the cathodic (*J*_p,c_) and anodic (*J*_p,a_) current densities as well as cathodic (*E*_p,c_) and anodic (*E*_p,a_) peak potentials for reduction of Fe(CN)_6_^3–^ and subsequent oxidation of Fe(CN)_6_^4–^. The electrochemical response of the bare gold electrode exhibits a clear voltammetric wave for the ferricyanide redox probe (Figure 5). In contrast, all of the LB film gold-modified electrodes (for the single, binary, and ternary systems studied here) show a significant suppression of the voltammetric wave accompanied by a shift in the cathodic peak, indicating partial blocking of the underlying metal electrode by the LB film.

**Table 2:** Parameters for the Cathodic (c) and Anodic (a) Processes of the Indicated Modified Gold Electrodes Using a Ferricyanide Redox Probe

monolayer	*J*_p,c_ (μA cm^–2^)	*E*_p,c_ (V)	*J*_p,a_ (μA cm^–2^)	*E*_p,a_ (V)
bare gold	292	0.181	290	0.282
DOPC	88	0.003	76	0.340
CsA	109	0.115	113	0.283
DOPC–CsA 0.50	115	0.099	129	0.290
DOPC–CsA–LG 0.25	100	0.145	118	0.282
DOPC–CsA–LG 0.50	88	0.130	108	0.283
DOPC–CsA–LG 0.75	85	0.125	92	0.282
LG	75	0.106	89	0.285

The DOPC monolayer on gold, Figure 5a, shows a large decrease in the cathodic voltammetric peak current density (*J*_p,c_), indicating that the monolayer significantly impedes the access of the redox probe, Fe(CN)_6_^3–^, to the underlying metal electrode. Moreover, the position of the cathodic peak is shifted to more negative potentials, which is a further indication of the difficulties of the redox probe to reach the electrode surface, revealing that the size of the pores is rather small and the redox probe follows an intricate path to penetrate the monolayer and reach the electrode surface. The subsequent oxidation peak of the Fe(CN)_6_^4–^ anions also exhibits considerable reduction in the current density compared with the bare electrode as well as deviation from the formal oxidation potential. These observations are consistent with a relatively well-packed monolayer that significantly inhibits the electron transfer between the redox probe in the solution and the underlying gold electrode. CsA (Figure 5a) and LG (Figure 5b) films also result in a decrease of the redox activity of the redox probe. Both *J*_p,c_ and *E*_p,c_ values for CsA and LG are indicative of the difficulties of the redox probe to reach the electrode surface, although the DOPC film is the one that better blocks the gold surface among the pure LB films studied here. The chemical structure of CsA, which as discussed before is preferentially packed in a vertical position with respect to the substrate, possibly favors the access of the redox probe to the gold surface in comparison with the more compact films incorporating either DOPC or LG. Moreover, the *E*_p,c_ value for the LG film (Table 2), which is not as shifted as the one for the DOPC film, indicates that the size of the pores or holes in the film is larger for this monolayer. Additionally, the partial and not total blocking of the electrode surface could be explained in terms of the fluid phase of the deposited films in which the alkyl chains are not fully packed.^31^

Mixed binary (DOPC–CsA 0.50) and ternary (DOPC–CsA–LG 0.25, 0.50, and 0.75) monolayers onto gold substrates were also studied by CV (Figure 5 and Table 2). The current densities for the binary DOPC–CsA 0.50 monolayer are the highest in the modified electrodes here studied. This result is consistent with the existence of repulsion interactions between DOPC and CsA molecules, which result in loosely packed films.^31^ The addition of LG to the DOPC–CsA system leads to a slight decrease in *J*_p,c_ and *J*_p,a_ in comparison to the DOPC–CsA films. Although this decrease is not drastic, it may suggest a lower presence of holes or pores in the deposited monolayers, which is in good agreement with the AFM images (Figure 4).

From these observations, it can be concluded that the DOPC film is the most compact layer in the one-component monolayers. On the contrary, for the ternary DOPC–CsA–LG monolayers, the strongest blocking effects are obtained for the monolayers with the LG molar fractions of 0.50 and 0.75, in agreement with the AFM images (Figure 4).

### 3.4 Wettability

**Figure 6:**
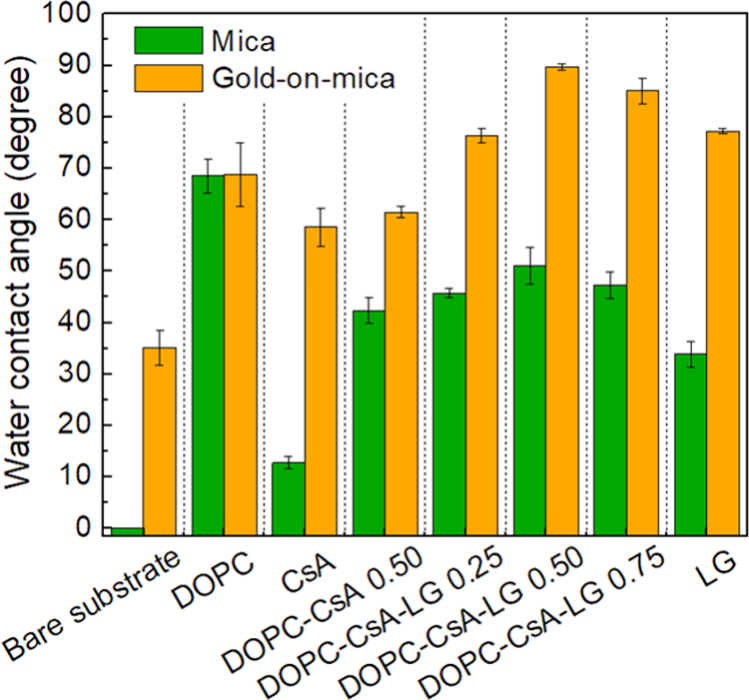
Contact angle values for the indicated single, binary, and ternary layers deposited on either mica or gold-on-mica substrates.

As mentioned above, biocompatible materials are those that being in contact with the tissue do not cause any unfavorable response of the organism. The wettability of the surface is closely related to such biocompatibility.^49^ Wettability is usually determined by measuring the contact angle (CA) of a drop of water on the material surface. A large water contact angle indicates small wettability or large hydrophobicity of the surface. As detailed below, significant differences in the surface coverage and film morphology on the two different substrates studied here are reflected in the film surface wettability. The water contact angles measured on the single, binary, and ternary systems deposited on either mica or gold-on-mica substrates are shown in Figure 6 (see also Figures S5 and S6, which contain the contact angle images).

The contact angle for a bare gold substrate is ca. 35° while that for mica is close to 0° (water spreads completely). The DOPC film onto the mica and gold substrates results in contact angles remarkably larger compared to the bare substrates. Interestingly, the DOPC-modified mica and gold substrates exhibit a comparable contact angle. This result indicates that DOPC forms a relatively compact layer with hydrocarbon chains sticking out and remarkably free of defects or holes. This observation is consistent with the AFM images shown in Figures 3 and 4 and also with the effective blocking of the gold-on-mica electrode (Figure 5). In contrast, for the other LB film-modified surfaces, the CA values are higher on the gold-supported layers in comparison with films deposited on mica, evidencing the influence of the underlying substrate on the physicochemical properties of the supported films. These observations are consistent with more permeable layers and even less flat surfaces in gold-supported layers. DOPC–CsA/gold and DOPC–CsA/mica substrates exhibit a CA difference of ca. 19°. Moreover, the difference between CA measured on CsA/gold and CsA/mica is about 45°.

These observations are interpreted here in terms of the repulsive interactions between DOPC and CsA molecules, which give rise to loosely packed films and even the presence of areas with a disruption of the order of the acyl chains. Therefore, as a result of a less compact film, a higher influence of the bare substrate in the measured CA for the DOPC–CsA system is observed. On the contrary, the addition of LG causes an increase in the contact angle as LG molecules are accommodated between DOPC and CsA molecules increasing the acyl chain density that prevents water penetration within the film. The most hydrophobic film in the series studied here is the DOPC–CsA–LG 0.50 monolayer (89.7 ± 0.6° on gold and 51.0 ± 3.6° on mica, Figure 6). The above observation correlates well with the improved homogeneity (lower RMS) of this LB film revealed by the AFM images and the strongest blocking effect demonstrated by the CV measurements. This result can be associated with the characteristic arrangement of the molecules in relation to each other. LG locates along DOPC but it is shifted toward its unsaturated bonds protecting them against oxidation. Owing to such an organization, the DOPC and LG hydrocarbon chains constitute a hydrophobic environment in which CsA can change from the open to the closed conformation and then penetrate the membrane hydrophobic core passively. This process is particularly important in terms of CsA release from the surface of a potential implant.^31^

Chemical heterogeneity of the film, interactions within the film constituents, and local changes in the film structure and/or roughness play a crucial role in the wetting properties of the surface. The differences in the surface topography, nanoscale roughness, and wettability of the substrates impose different hydrophobicity for the obtained layers. The above surface parameters affect the adhesion strength and the lateral interactions of adjacent molecules, which results in alterations of the overall packing and arrangement of molecules. A slightly rougher gold-on-mica surface may provoke a looser packing of the films. Such loosely packed structures are more permeable to water, which may have access to the underlying substrate. Hence, its surface properties contribute to the total wettability of the film. Since the gold-on-mica surface is less hydrophilic (CA ≈ 35°) than that of mica (CA ≈ 0°), the contact angles measured for the LB films deposited on the gold substrate are higher. Nevertheless, according to AFM images (Figures 3 and 4) and RMS values, homogeneous films are obtained for both substrates. Taking into account the range of the contact angle values measured here (Figure 6), the DOPC–CsA–LG layers on gold-on-mica can be classified as weakly hydrophobic (90° > CA > 56–65°), whereas the DOPC–CsA–LG layers on mica as weakly hydrophilic (56–65° > CA > 0^o^).^50^ Regardless of the substrate, this wettability is intermediate between highly hydrophilic and highly hydrophobic.

When the biomaterial is in contact with blood, platelets respond differently to hydrophobic or hydrophilic surfaces.^49,51^ Additionally, some results have demonstrated that the activation of platelets decreases with increasing surface wettability.^49,52^ On the other hand, biomaterials characterized by a small contact angle, and therefore a large surface wettability, do not necessarily exhibit increased biocompatibility. Highly hydrophobic surfaces enlarge cell affinity, while highly hydrophilic surfaces hinder cell–cell interactions. Therefore, blood-contacting biomaterials should maintain a balance between the hydrophobic and hydrophilic domains on the surface to reach the best compatibility.^35,49,53^ Thus, the solid-supported DOPC–CsA–LG monolayers with moderate hydrophobicity/hydrophilicity seem to be advantageous for cell adhesion and proliferation. As candidates for hemocompatible biomaterials in various biomedical applications, these surfaces can be qualified for further *in vitro* experiments.

## 4 Conclusions

LB films formed by DOPC, CsA, and/or LG on mica as well as on gold (gold on mica, on glass, or on quartz) were investigated by means of quartz crystal microbalance (QCM), atomic force microscopy (AFM), cyclic voltammetry (CV), and contact angle (CA) measurements. QCM measurements revealed that molecules in the LB films are tilted with respect to the normal surface, which is consistent with the monolayers being transferred at 10 mN m^–1^, i.e., in a liquid-expanded (LE) state at which they are loosely packed, reflecting the fluid state of the natural membranes. Despite the fluid nature of these films at the target surface pressure of transference, such surface pressure has been proved to be high enough to ensure sufficient cohesion in the monolayers for their effective transference onto gold and mica substrates as evidenced by the transfer ratio (TR) values. TR higher than 1 was related to a more vertical orientation of the molecules upon the deposition induced by the substrate nature and/or a change in the interactions between molecules governed by specific interactions with the substrate surface.

The analysis of the AFM images shows that the LB films on mica and gold substrates are very smooth (RMS at the nanometer level) albeit the RMS roughness of the gold-on-mica-supported LB films is slightly higher due to the steps and terraces typical for the topography of this substrate. Only for DOPC–CsA 0.50, the presence of areas with a disruption of the order of the acyl chains was found. These observations were further supported by CV experiments, which revealed partial blocking of the gold electrode surface by the monolayers with the presence of holes or pores in the films. Nevertheless, the DOPC film was found to be the most compact among the single monolayers, while in the case of the mixed LB films, the strongest blocking effects were observed for the DOPC–CsA–LG monolayers with the LG molar fractions of 0.50 and 0.75. Importantly, at the same proportion of components, the highest surface coverage was achieved, confirming the formation of more packed films with reduced permeability to water as evidenced by the increased values of contact angles. All of these findings correlate well with the improved homogeneity (lower RMS) of these LB films revealed by the AFM images. This result has been interpreted in terms of the favored attractive interactions between molecules at these molar ratios, i.e., LG molecules act as a linker between those of DOPC and CsA that favors miscibility through hydrogen bonding and Lifshitz–van der Waals forces.

From the observed surface characteristics of multicomponent DOPC–CsA–LG 0.50 and 0.75 monolayers, we conclude that the use of the LB technique ensures a good miscibility of the components induced upon the compression process, resulting in high-quality films with enhanced physicochemical properties. Therefore, these films could be suitable in biocoating applications for implant biocompatibility and proper functioning within the living organism. This type of fundamental research represents the first screening for a rational and scientific approach to design biocompatible coatings containing biologically active compounds, opening up new perspectives for cardiovascular implants.

## Supporting Information Available

Langmuir monolayer stability (Figures S1 and S2) and Langmuir–Blodgett deposition process on mica and on gold substrates at the surface pressure of 10 mN m^–1^ (Figure S3 and Table S1); gold surface coverage of the QCM resonator expressed in molecules cm^–2^ (Figure S4); and contact angle images for the LB films on mica (Figure S5) and gold (Figure S6) (PDF)
