# Supplementary material for: Characteristics of Phospholipid–Immunosuppressant–Antioxidant Mixed Langmuir–Blodgett Films
Source: J Phys Chem B. 2022 Sep 6;126(36):6936–47. doi: 10.1021/acs.jpcb.2c03300 (PMC9483916; doi:10.1021/acs.jpcb.2c03300)
Supplement: Supplementary file 1 — jp2c03300_si_001.pdf [file jp2c03300_si_001.pdf]

# Characteristics of Phospholipid-Immunosuppressant-Antioxidant Mixed Langmuir-Blodgett Films

Małgorzata Jurak,[a*] Klaudia Szafran[a], Pilar Cea[b,c,d] and Santiago Martín[b,c,d]

[a] Department of Interfacial Phenomena, Institute of Chemical Sciences, Faculty of Chemistry, Maria Curie-Skłodowska University, 20031 Lublin, Poland
[b] Instituto de Nanociencia y Materiales de Aragón (INMA), CSIC-Universidad de Zaragoza, 50009 Zaragoza, Spain
[c] Departamento de Química Física, Facultad de Ciencias, Universidad de Zaragoza, 50009 Zaragoza, Spain
[d] Laboratorio de Microscopias Avanzadas, LMA, C/Mariano Esquilor s/n 50018, Zaragoza

*__Corresponding author__ (M. Jurak)
e-mail address: malgorzata.jurak@mail.umcs.pl
phone: +48815375547, fax: +48815375656

## 1. LANGMUIR MONOLAYER STABILITY AND LANGMUIR-BLODGETT DEPOSITION

The stability of the monolayers at a constant surface pressure of 10 mN m$^{-1}$ was evaluated by recording the change in the area per molecule (with barriers moving at a speed of 5 mm min$^{-1}$). The results are shown in Figure S1. DOPC shows a significant change in the area per molecule revealing a poor stability. In contrast, CsA, LG as well as the binary and ternary mixtures are more stable. The largest area reduction for pure DOPC (46 %) and DOPC-containing mixed monolayers (13-17 %) indicates the desorption of molecules from the air-water interface. The reason for that can be a partial oxidation and/or decomposition of the DOPC molecules exposed to the ambient atmosphere.[23] These processes can be inhibited by the presence of other compounds (CsA and/or LG) in the mixed films.

Additionally, the surface pressure fluctuations with time (0-60 min) was also recorded by maintaining a target pressure of 10 mN m$^{-1}$ and moving the barriers at a speed of 5 mm min$^{-1}$. These results are presented in Figure S2. Such fluctuations can be considered negligible (maximum deviations of 0.15 mN m$^{-1}$) revealing that a barrier speed of 5 mm min$^{-1}$ is an appropriate parameter in these experiments to ensure a constant surface pressure during the transference process.

Accordingly, the monolayer transfer was carried out by a vertical dipping (emersion) procedure at a rate of 5 mm min$^{-1}$ immediately after reaching the target pressure. This strategy was aimed to prevent the undesirable effect of loss of molecules due to exposing the monolayers floating on the water surface to prolonged contact with air.

The obtained transfer ratio ($TR$) plots when a mica substrate was used were determined as a decrease in the monolayer surface area on the subphase ($\Delta A_m$) divided by the substrate coated area ($A_s$) (Figure S3). Due to the instability of the monolayers (Figure S1) the transfer ratio ( $TR$) values determined from the Langmuir trough software (Figure S3) were corrected for the decrease of the monolayer surface area related to the loss of molecules in the time needed to transfer the film (Table S1). Whilst for a gold substrate these were calculated by dividing the area per molecule for each Langmuir film ($A_i$) at the transference pressure (10 mN m$^{-1}$) per the surface coverage ($A_e$) determined by measures of the QCM for each film (Table S1). The $A_e$ values were calculated as the reciprocal of the coverage expressed in molecules cm$^{-2}$ (Figure S4).

Therefore, all monolayers were successfully transferred to mica or gold substrates even at such a low surface pressure. However, as it can be observed in Table S1, some transfer ratio values are much greater than 1. This indicates that more molecules than those expected for the monolayer have been deposited on the substrate.

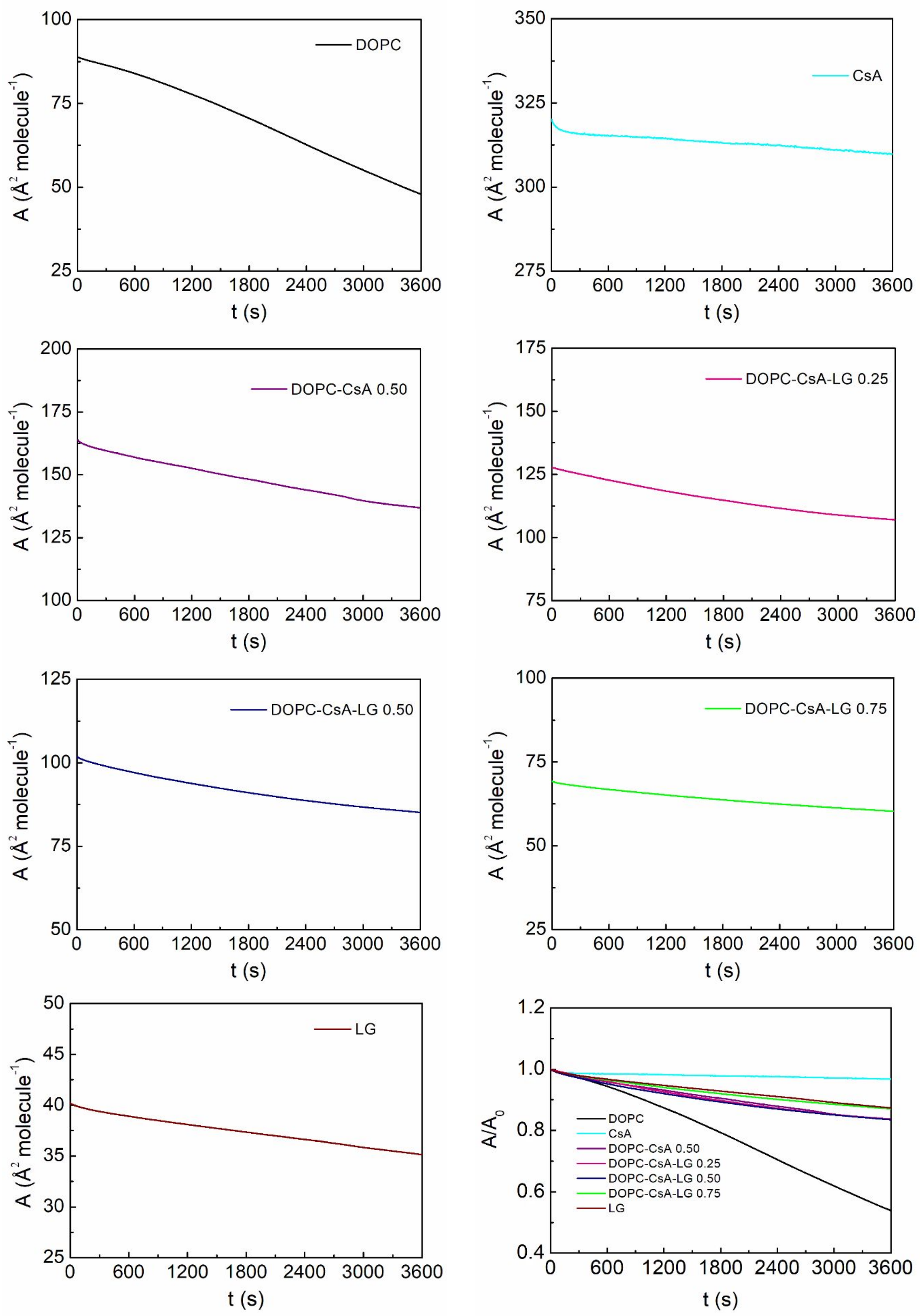


**Figure S1.** Area per molecule versus time $(A - t)$ changes during 1 hour stabilization at 10 mN m$^{-1}$ with the barrier speed of 5 mm min$^{-1}$. The last figure shows the relative molecular area $(\frac{A}{A_0})$ changes with time.

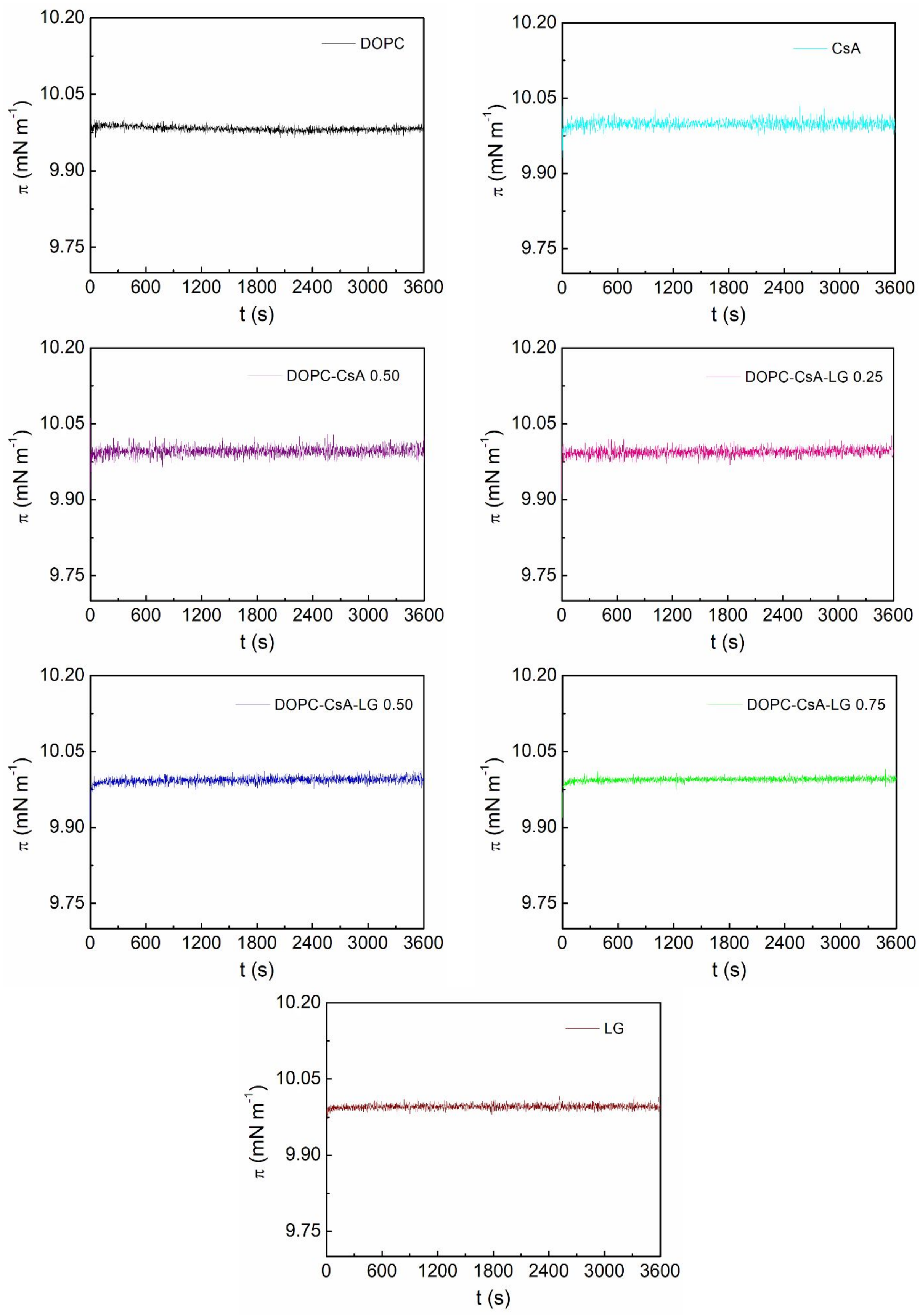


**Figure S2.** Surface pressure versus time ($\pi - t$) changes during 1 hour stabilization at 10 mN m$^{-1}$ with the barrier speed of 5 mm min$^{-1}$.

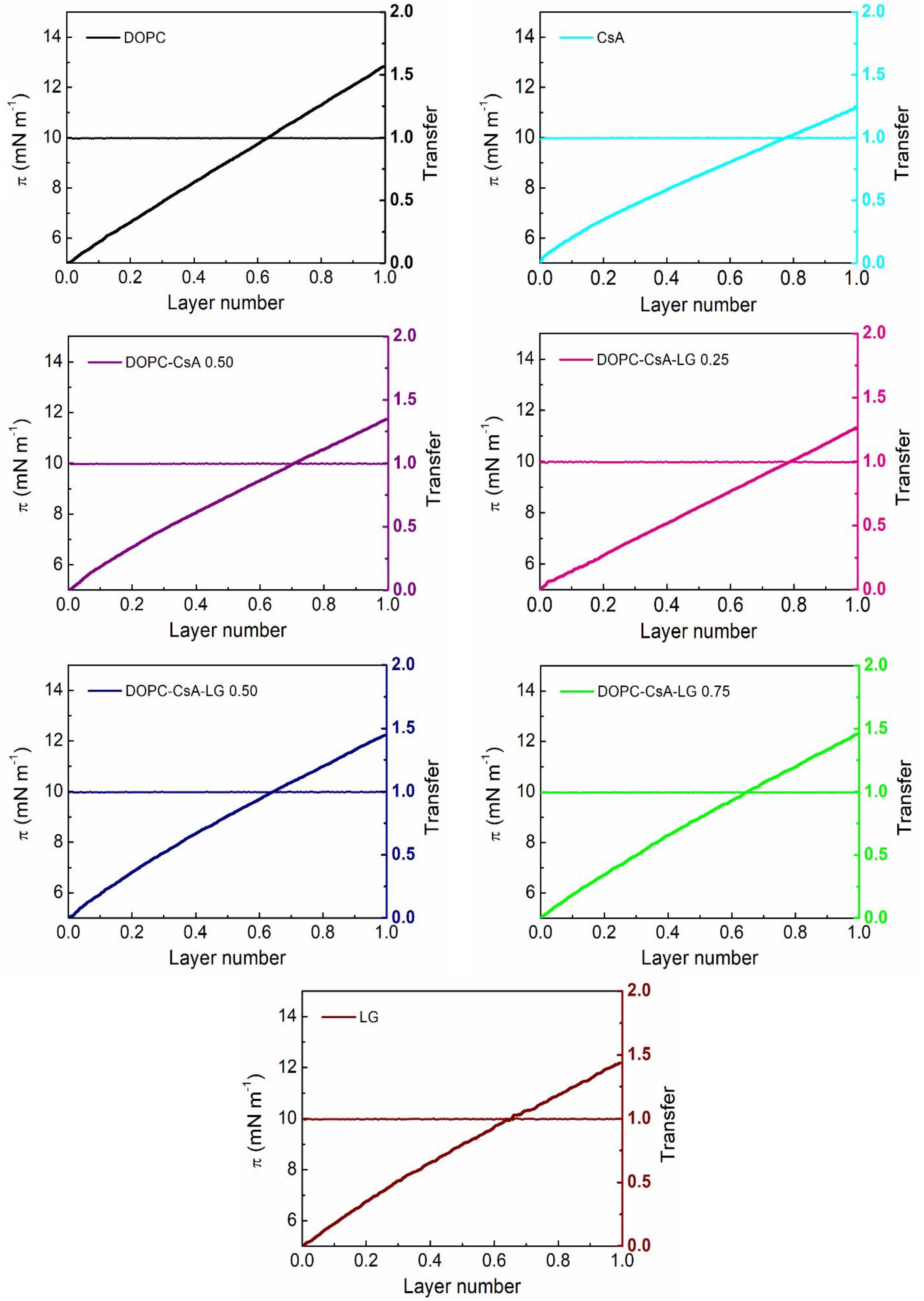


**Figure S3.** Transfer ratio plots ($Transfer - Layer\ number$) during Langmuir-Blodgett deposition onto mica at 10 mN $m^{-1}$ with the barrier speed of 5 mm $min^{-1}$. The transference was carried out immediately after reaching the target pressure.

**Table S1.** Characteristic parameters for the monolayers: compression modulus ($C_S^{-1}$), surface potential changes ($\Delta V$), mean area per molecule ($A_i$), determined from $\pi - A$ and $\Delta V - A$ isotherms as well as transfer ratio ($TR$) values for the deposition process on gold and mica at 10 mN m$^{-1}$.

| **Monolayer** | $C_S^{-1}$ **(mN m$^{-1}$)** | $\Delta V$ **(mV)** | $A_i$ **(Å$^2$)** | $TR = \frac{A_i}{A_e}$ **gold** | $TR = \frac{\Delta A_m}{A_s}$ **mica** |
|---|---|---|---|---|---|
| DOPC | 48 | 278 | 78.4 | 0.8 ± 0.1 | 0.8 ± 0.3 |
| CsA | 66 | 249 | 210.1 | 1.1 ± 0.2 | 1.0 ± 0.1 |
| DOPC-CsA 0.50 | 53 | 340 | 156.2 | 1.3 ± 0.1 | 1.0 ± 0.2 |
| DOPC-CsA-LG 0.25 | 41 | 302 | 116.9 | 1.2 ± 0.2 | 0.9 ± 0.3 |
| DOPC-CsA-LG 0.50 | 45 | 224 | 88.2 | 1.8 ± 0.2 | 1.1 ± 0.3 |
| DOPC-CsA-LG 0.75 | 41 | 233 | 63.3 | 1.1 ± 0.2 | 0.9 ± 0.3 |
| LG | 30 | -26 | 37.5 | 0.9 ± 0.1 | 0.9 ± 0.2 |

***Note:*** *Compression modulus ($C_S^{-1}$), surface potential changes ($\Delta V$) and mean area per molecule (A) were taken from our previous paper.*[31]

## 2. GOLD SURFACE COVERAGE OF THE QCM RESONATOR

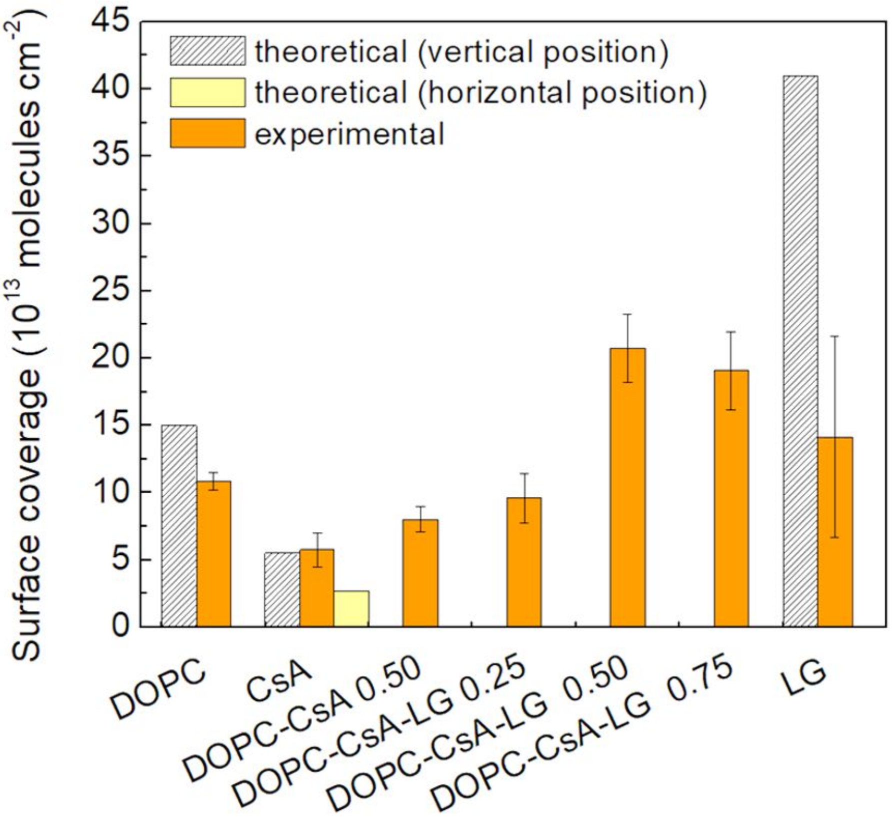


**Figure S4**. Surface coverage expressed in molecules $cm^{-2}$ for the indicated single, binary and ternary monolayers as well as the theoretical surface coverage determined taking into account a vertical or horizontal (CsA) arrangement of the molecules.

## 3. CONTACT ANGLES (CA) IMAGES

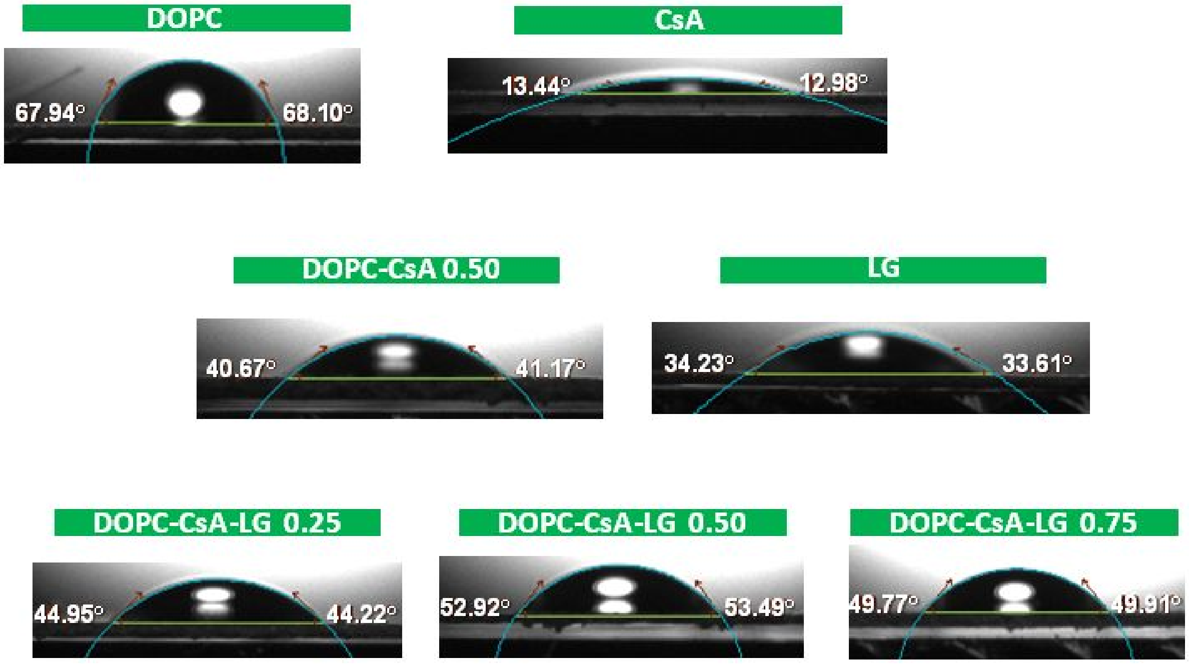


**Figure S5.** Contact angle (CA) images for the indicated one-layer LB films transferred at 10 mN $m^{-1}$ onto mica.

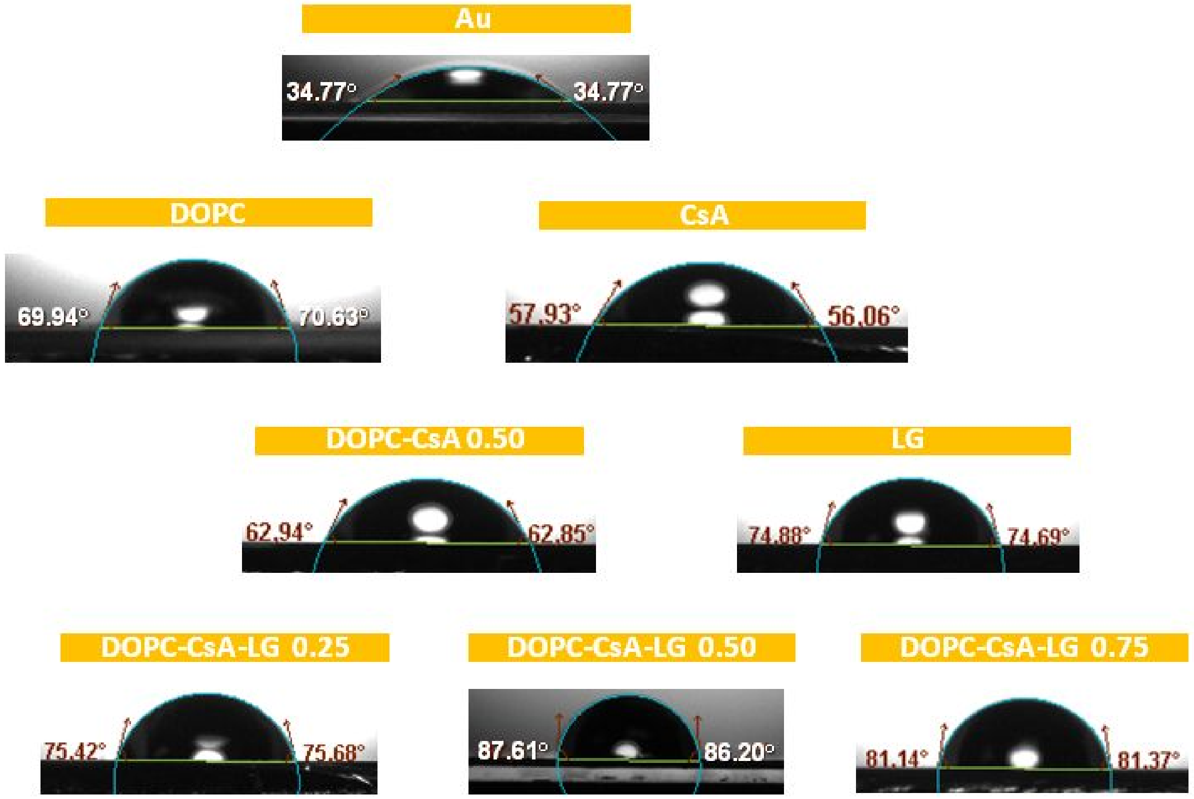


**Figure S6.** Contact angle (CA) images for the indicated one-layer LB films transferred at 10 mN $m^{-1}$ onto gold.
